# Characterization of Overwintering Sites of the Invasive Brown Marmorated Stink Bug in Natural Landscapes Using Human Surveyors and Detector Canines

**DOI:** 10.1371/journal.pone.0091575

**Published:** 2014-04-09

**Authors:** Doo-Hyung Lee, John P. Cullum, Jennifer L. Anderson, Jodi L. Daugherty, Lisa M. Beckett, Tracy C. Leskey

**Affiliations:** 1 U.S. Department of Agriculture – Agricultural Research Service, Appalachian Fruit Research Station, Kearneysville, West Virginia, United States of America; 2 Department of Entomology, Virginia Tech, Winchester, Virginia, United States of America; 3 U.S. Department of Agriculture – Animal and Plant Health Inspection Service, National Detector Dog Training Center, Newnan, Georgia, United States of America; Natural Resources Canada, Canada

## Abstract

*Halyomorpha halys* is an invasive species from Asia causing major economic losses in agricultural production in the mid-Atlantic region of the United States. Unlike other crop pests, *H. halys* is also well-known for nuisance problems in urban, suburban, and rural areas, as massive numbers of adults often invade human-made structures to overwinter inside protected environments. Research efforts have focused on populations in human-made structures while overwintering ecology of *H. halys* in natural landscapes is virtually unknown. We explored forested landscapes in the mid-Atlantic region to locate and characterize natural overwintering structures used by *H. halys.* We also evaluated the use of detector canines to locate overwintering *H. halys* to enhance the accuracy and efficiency of surveys. From these studies, we indentified shared characteristics of overwintering sites used by *H. halys* in natural landscapes. Overwintering *H. halys* were recovered from dry crevices in dead, standing trees with thick bark, particularly oak (*Quercus* spp.) and locust (*Robinia* spp.); these characteristics were shared by 11.8% of all dead trees in surveyed landscapes. For trees with favorable characteristics, we sampled ∼20% of the total above-ground tree area and recovered 5.9 adults per tree from the trees with *H. halys* present. Two detector canines were successfully trained to recognize and detect the odor of adult *H. halys* yielding >84% accuracy in laboratory and semi-field trials. Detector canines also found overwintering *H. halys* under field conditions. In particular, overwintering *H. halys* were recovered only from dead trees that yielded positive indications from the canines and shared key tree characteristics established by human surveyors. The identified characteristics of natural overwintering sites of *H. halys* will serve as baseline information to establish crop economic risk levels posed by overwintering populations, and accordingly develop sustainable management programs.

## Introduction

Invasive species in the United States cause major environmental and economic losses in agroecosystems, and the number of foreign species continues to increase [1]. Brown marmorated stink bug, *Halyomorpha halys* (Hemiptera: Pentatomidae), is an invasive species from Asia [2], first discovered in the United States in the mid-1990s [3]. This invasive species has been detected in 40 states and the District of Columbia as of 2013 (www.stopbmsb.org), Canada [4], Switzerland [5], Liechtenstein [6], Germany [7], and France [8]. *H. halys* has emerged as a key pest in agriculture and created major nuisance problems especially in the mid-Atlantic region of the United States [9]. Because *H. halys* is a newly established pest, management tactics against this species are often limited to repeated insecticide applications. For instance, some tree fruit growers in the mid-Atlantic region increased the number of insecticide applications made to their orchards nearly four-fold in 2011 due to *H. halys* pest pressure, following the first outbreak of this pest in 2010 in the region [10].

This insect overwinters as an adult and there are typically one or two generations per year in the mid-Atlantic region. During the growing season, both adults and nymphs can cause widespread destructive feeding injury to a variety of crops [9], [10]. Because both adults and nymphs are very mobile, moving among different host species throughout the growing season [11], [12], it is difficult to monitor the populations and apply management tactics accordingly. Because of these complex movement patterns, overwintering locations can serve as a fixed location for monitoring and provide reliable information as a measure of risk of *H. halys* dispersal into agricultural fields. Moreover, overwintered adults are significantly more susceptible to insecticides compared with F_1_ adults later in the season [13]. Therefore, targeted management of the overwintering *H. halys* population before they disperse into agricultural production could provide an effective means to reduce establishment of field populations and mitigate risk in the later season. For this reason, understanding of overwintering ecology of *H. halys* is fundamental to the development of sustainable management programs for this invasive species.

*H. halys* overwinter in sheltered, protected locations. Unlike many other agricultural pests, this species is well-known for nuisance problems, as massive numbers of adults often invade and overwinter inside human-made structures [14]–[17]. Perhaps because of this highly apparent movement and appearance on and inside human-made structures, the overwintering ecology of *H. halys* in natural landscapes has remained virtually unexplored. Only a few articles describe overwintering ecology in its native range [2] and report in isolated and general observations that adults can hibernate in ground litter and inside tree holes [18], under tree bark [19], and in dry, high-elevation mountains [11]. Currently, characteristics of natural overwintering sites of *H. halys*, the abundance of the insects in those sites, and the risk posed by these populations to cultivated crops remain unknown.

Anecdotal evidence supports that *H. halys* populations disperse into cultivated crops from wooded areas with few human-made structures in the vicinity. These observations indicate that further study of *H. halys* populations overwintering in natural landscapes and the risk that the populations pose to agricultural production as a ‘pest reservoir’ is warranted. In particular, given that adult *H. halys* are very mobile and capable of dispersing >2 km in a mark-release-recapture study [20] and in a tethered flight test [Wiman, N.G., unpublished data; Lee, D.-H., unpublished data], *H. halys* populations overwintering in forested areas are likely significant but unchecked threats to agriculture.

However, sampling overwintering insects in natural landscapes is often challenging due to the amount of time and resources required to survey small and concealed individuals in the context of complex environments. Indeed, this challenging distribution pattern is a major factor limiting legitimate risk analysis on *H. halys* populations in natural landscapes. Domestic canines, *Canis familiaris* L., have exceptional ability to detect target scents from biotic and abiotic materials, increasing sampling accuracy can efficacy substantially. In this study, we evaluated potential use of detector canines in locating *H. halys* populations overwintering in natural landscapes to enhance the accuracy and efficiency of sampling efforts. Based on data sets collected by human surveyors and detector canines, we report and quantify for the first time the overwintering sites used by *H. halys* populations in natural landscapes in the invaded region.

## Materials and Methods

### Study sites

Sampling aimed at studying *H. halys* populations overwintering in the natural landscape were conducted in wooded areas in the winter months of 2011–2012 and 2012–2013. Study sites were established in two types of deciduous woodlands in Maryland and West Virginia: 1) woodlots directly adjacent to agricultural production areas and 2) mountainous sites on the Appalachian Trail (MD), Cunningham Falls State Park (MD), and the Shannondale Springs Wildlife Management Area (WV). Research permits were obtained from Maryland Department of Natural Resources, West Virginia Division of Natural Resources, and the University of Maryland to conduct sampling in those forested areas. These woodland areas are typical of the mid-Atlantic region with mixed oaks and northern hardwoods, often even-aged stands comprised of second growth. Dead trees and leaf litter were selected for sampling based on known natural overwintering sites of pentatomid species [19], [21], [22].

### Strip transect samples by human surveyors

Strip transect sampling was conducted to establish if *H. halys* overwinter within dead trees and to characterize all dead trees in the area surveyed (Table 1). In each study site, 500-m^2^ (50×10 m) sample areas were established with distances of >30 m between strip transects depending on the topographical configuration of the site. The following characteristics were recorded for all sampled dead trees: 1) species, 2) diameter at breast height (DBH), 3) estimated height, and 4) position (standing vs. downed). Trees with DBH of <9.5 cm were excluded from sampling. A total of 980 dead trees were destructively sampled in 75 transects (37,500-m^2^ total area) over the two seasons to record the presence or absence of overwintering *H. halys* (Table 1). Sampling for *H. halys* was conducted by carefully peeling back tree bark and excavating decomposed tree tissue using small axes. Fixed sampling time per tree was determined and used based on DBH: 1) 4.0 min per person for 10–15 cm (DBH); 2) 6.5 min for 16–21 cm; 3) 9.0 min for 22–27 cm; and 4) 11.5 min for >28 cm. Generally, standing trees were sampled up to ∼2.5 m from the base; downed trees were sampled across the entire trunk and to the upper canopy if time allowed.

**Table 1:** List of sample sites, number of dead trees, and detection of overwintering *Halyomorpha halys* in the dead trees.

Year	Sample site^1^	Geographic coordinates	Sample site size (m^2^)^2^	No. of transects	No. of trees found	No. of trees sampled^3^	No. of trees with *H. halys*
2011–2012	AFRS1	39°21′20.0″N, 77°53′23.5″W	25,300	3	45	45	0
	AFRS2	39°21′23.1″N, 77°53′11.4″W	26,100	3	40	40	2
	AFRS3	39°20′42.7″N, 77°53′51.4″W	112,000	6	94	94	2
	KV1	39°30′59.9″N, 77°44′01.7″W	43,200	4	57	57	0
	KV2	39°30′20.4″N, 77°43′41.3″W	193,000	8	110	110	3
	AT1	39°24′01.7″N, 77°38′29.3″W	n/a	6	90	90	0
	AT2	39°24′34.9″N, 77°38′24.8″W	n/a	4	67	16^*^	0
	AT3	39°30′13.3″N, 77°37′18.3″W	n/a	4	94	20^*^	0
	AT4	39°30′29.7″N, 77°37′21.7″W	n/a	2	43	8^*^	0
	AT5	39°30′40.9″N, 77°37′14.1″W	n/a	3	78	32^*^	5
	AT6	39°30′56.0″N, 77°37′10.4″W	n/a	4	73	17^*^	2
2012–2013	AFRS3	39°20′42.7″N, 77°53′51.4″W	112,000	6	73	73	1
	KV2	39°30′20.4″N, 77°43′41.3″W	193,000	7	77	77	3
	AT7	39°32′34.6″N, 77°35′53.0″W	n/a	4	75	75	2
	AT8	39°32′49.5″N, 77°35′45.4″W	n/a	4	78	78	0
	CFSP1	39°34′05.8″N, 77°28′13.3″W	n/a	3	105	105	0
	CFSP2	39°32′31.2″N, 77°29′22.3″W	n/a	4	43	43	1

1 AFRS refers to the research farm of the USDA-ARS, Appalachian Fruit Research Station in Kearneysville, WV; KV refers to the research farm at the University of Maryland Research Station in Keedysville, MD; AT refers to the Appalachian Trail, MD. CFSP refers to the Cunningham Falls State Park, MD.

2 n/a indicates that sample sites were located in continuous mountainous areas.

3 Asterisk indicates that only standing trees were destructively sampled.

A separate selective sampling was conducted to validate the characteristics of dead trees that we had established as favorable for overwintering sites based on the results of random samples from strip transects described above. From these trees, we also estimated the density of overwintering *H. halys*. Fifty and 54 trees were selected in 2011–2012 and 2012–2013, respectively, and examined up to ∼3.0 m above the base (∼20% of the total tree area) until all accessible potential overwintering sites were excavated by peeling away bark and using small axes.

For data analysis, the proportions of trees harboring *H. halys* were compared between tree characteristics (e.g., standing vs. downed), sampling years, and sampling methods using the likelihood ratio test or the Fisher's exact test (JMP Genomics 5.0, SAS Institute). The mean numbers of *H. halys* were compared between sampling years using the Wilcoxon/Kruskal-Wallis test due to the non-normality of the data (JMP Genomics 5.0, SAS Institute).

### Leaf litter samples by human surveyors

Leaf litter was sampled from wooded areas using 1×1-m grids placed on the ground. Grids were laid out randomly in the study sites with >20-m distances between the samples. All leaf litter material in the grid (∼5.0-cm depth) was visually inspected for the presence of *H. halys* and other pentatomid species. A total of 120 samples were processed in the field during each year of the two winter seasons.

### Ethics statement

Canines used in the scent detection trials are or were property of United States Department of Agriculture (USDA). Canines housed at the USDA National Detector Dog Training Center (NDDTC) (Newnan, GA) are trained for various scent detection programs, and are not utilized in experimental procedures. Canine care is compliant with or exceeds USDA Guidelines and Standards for housing, feeding and care.

### Detector canines and data collection

In November 2012 and January 2013, two detector canines, ‘Opal’ (Canine 1) and ‘Tig’ (Canine 2), respectively, were assigned to the detection program for *H. halys*. Both canines were ∼3-yr old Labrador retrievers housed and trained at the NDDTC. They were procured from a shelter in the greater Atlanta area at approximately the same time. Both canines had been assigned to the NDDTC's mollusk detection program for ∼1 yr before being trained to detect *H. halys*. The NDDTC utilized operant conditioning when training all of its detector canines. The canines were trained to give a final response, “sit”, in the presence of a target odor. Once canines successfully paired the final response with the target odor, the canines were trained to search for the target odor in increasingly complex scenarios by reinforcing desired responses. Canines earned a food reward for successfully searching for and responding to the presence of target odor. Canines were trained to pinpoint a source of odor with their nose when given the command “show me”. This behavior was used to help identify the source of a target odor that might not be easily identified by the handler.

The data generated by detector canine studies are presented as proficiency levels. Proficiency levels are based on detector canines correctly indicating presence of *H. halys* and not responding to blank controls across the trials tested. Responses by canines were interpreted by handlers and defined as positive indications when the canines sat next to test odor. Handlers were responsible for interpreting and following up on changes in canine behavior (e.g., increased sniffing, head snaps, and changes in ear position or tail movement). Proficiency of responding to *H. halys* was calculated by dividing the number of correct positive indications of the target odor by the total number of *H. halys* targets in the trials. Likewise, proficiency of not responding to blank controls was calculated by dividing the number of correct non-response to the blanks by the total number of blank controls in the trial.

### Laboratory detection of *H. halys* by canines

Training was conducted in a room (∼20°C) at the NDDTC. In this indoor study, a target odor was prepared by placing five *H. halys* adults in a paper containment bag (6×8 cm); empty bags were used as blank controls. The target or blank containment bags were concealed inside cardboard boxes (30.5×30.5×30.5 cm) and three ventilation holes were made on the box. In each trial, a total of 25 boxes were arranged 2 m apart in a square formation. Among 25 boxes, 1–5 boxes included target odors. *H. halys* were allowed to settle in containment bags for ∼3 h before initiation of a trial. Positions of the boxes were randomized between trials. In each trial, both canines were tested under the same experimental design. All 25 boxes were examined by two detector canines (Video S1). For each canine, a total of 18 trials were conducted during which 450 boxes were examined. Of these, 50 boxes contained *H. halys* targets and 400 served as blank controls. Human handlers did not know the location of target odors or blank controls for any trial. In the trials, positive indications by canines (i.e., sitting) on the boxes were recorded and checked whether or not they were correct.

### Semi-field detection of *H. halys* by canines

**Figure 1:**
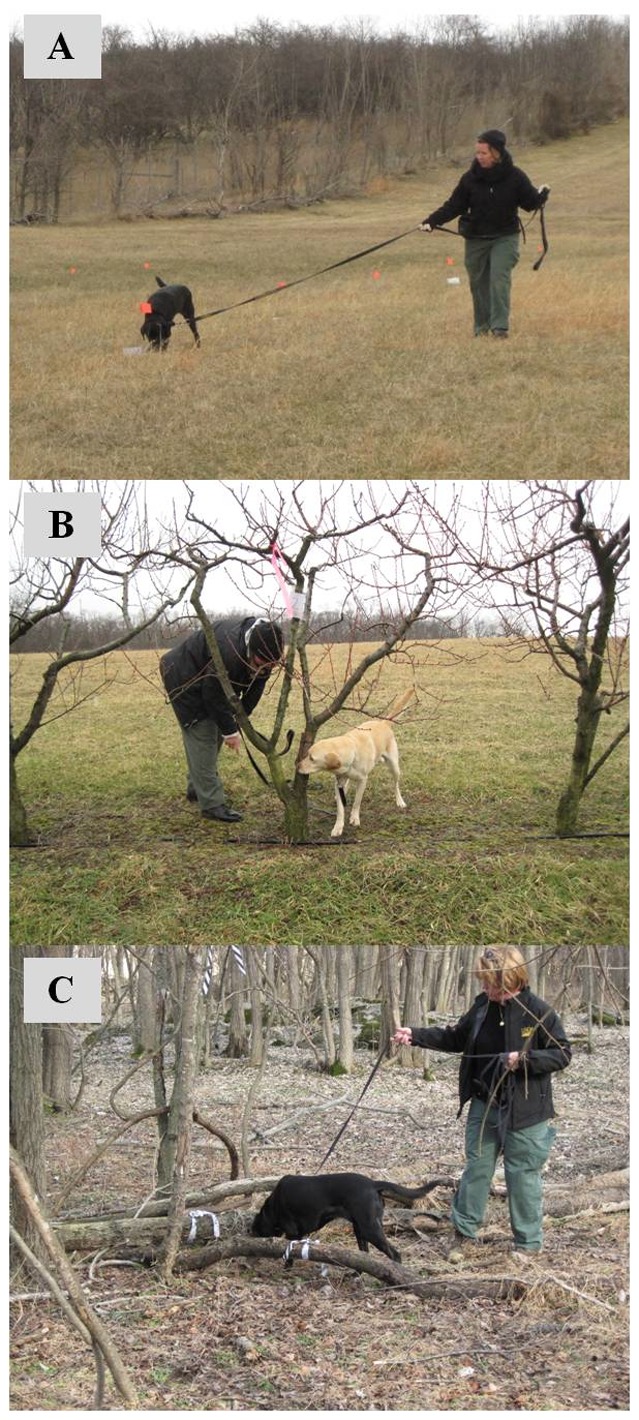
Canine detection of *Halyomorpha halys* concealed on the ground (A), concealed on the live peach trees (B), and naturally existing in the natural landscapes (C).

A semi-field study was conducted in an open grassy plot with relatively uniform terrain at the Appalachian Fruit Research Station (AFRS), USDA-ARS, Kearneysville, WV (39°21′29.99″N; 77°53′25.44″W) (Fig. 1A). This open field provided a less complex searching environment and facilitated transition from an indoor laboratory setting to outdoor field environments. In each trial, a total of 20 odor point sources were established 5 m apart on the ground. Each odor point had a paper containment bag with two live *H. halys* adults inside or an empty bag. All bags were concealed under a piece of locust tree bark (15×20 cm). Among the 20 points, 2–4 locations included *H. halys* targets; all others included empty containment bags as blank controls. Locations of *H. halys* targets were randomized between trials. *H. halys* were allowed to settle in containment bags for >2.5 h before initiation of a trial. All odor point sources were examined by two detector canines and the survey path was modified by human handlers depending on wind direction. A total of 13 trials were conducted for each canine during which 260 points were examined. Of these, 40 points contained *H. halys* targets and 220 served as blank controls.

A second semi-field experiment was conducted in an isolated peach plot (55×55 m) with 10 rows of peach trees (∼25 trees per row) at the AFRS (39°21′26.55″N; 77°53′21.74″W) (Fig. 1B). In general, the experimental setting and methods for this experiment were similar to the above experiment conducted in an open field. Odor point sources were established in this cultivated plot of live trees in which overwintering *H. halys* were not pre-existing. This experimental plot provided a more complex searching environment compared with the previous experiments. In each trial, a total of 20 odor points were established at ∼0.8 m above the ground on the peach tree trunks (one odor point source per tree) in the two tree rows. Among 20 points, 2–3 locations included *H. halys* targets and their locations were randomized between trials. A total of 10 trials were conducted for each canine during which 200 odor points were examined. Of these, 26 points contained *H. halys* targets and 176 served as blank controls.

### Detection of *H. halys* by canines in natural landscape

This study was conducted in a woodlot surrounded by agricultural production near the AFRS (39°20′42.18″N; 77°53′51.73″W) (Fig. 1C). A total of 12 strip transects (50×10 m/transect) were randomly laid out with distances of >20 m between transects in the woodlot. All dead trees found within strip transects were marked prior to surveys being conducted. There were a total of 187 dead trees across all transects. Each dead tree was examined by two detector canines over four days. When detector canines exhibited positive indications to dead trees, those trees were destructively sampled by peeling away bark and using small axes during the survey to verify canine responses. After the survey, 50 additional dead trees that canines did not show positive indications were randomly chosen and destructively sampled to verify the canine responses.

## Results

### Strip transect samples by human surveyors

**Figure 2:**
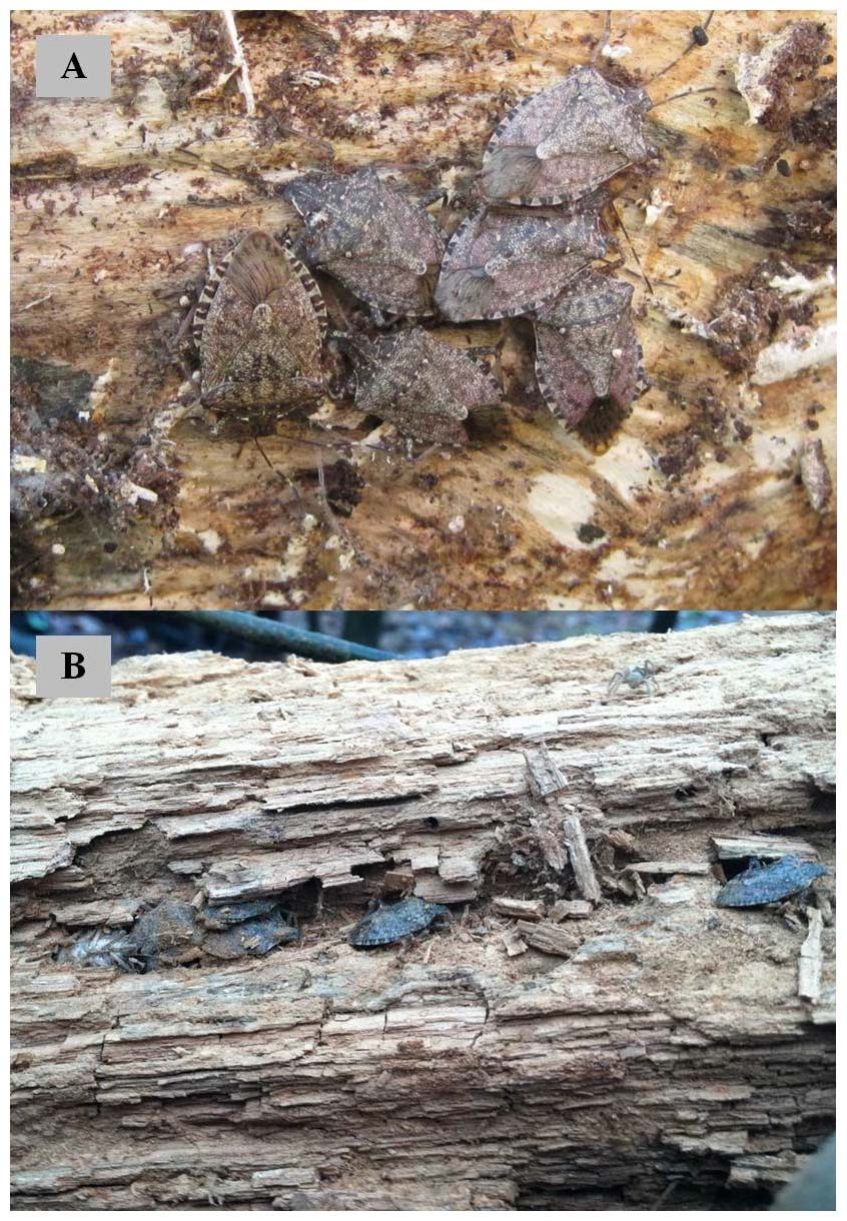
Overwintering *Halyomorpha halys* found under the tree bark (A) and inside the decomposed tree tissue.

**Figure 3:**
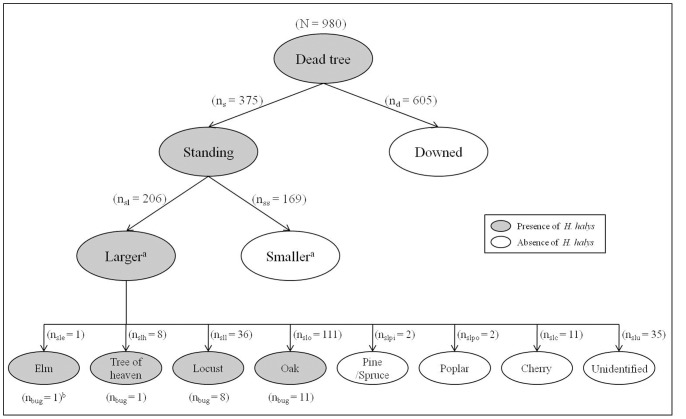
Characterization of dead trees used by *Halyomorpha halys* for overwintering sites in the forested areas identified by human surveyors. Numbers in the parentheses are the number of dead trees with given characteristics. ^a^Larger refers to trees with diameter at breast height (DBH) >19 cm; smaller refers to tree with DBH ≤19 cm. n_bug_ = number of trees harboring *H. halys*.

Overwintering *H. halys* were found from 14 of the 529 dead trees and from 7 of the 451 dead trees sampled in 2011–2012 and 2012–2013, respectively (Table 1). There was no significant difference in the proportions of trees harboring *H. halys* between the two years (*χ* ^2^ = 0.004, d.f. = 1, P = 0.949). *H. halys* were found between loose tree bark and the trunk (Fig. 2A) and inside decomposed woody tissue (Fig. 2B). Trees that harbored overwintering *H. halys* shared three characteristics (Fig. 3). First, *H. halys* were located only in standing trees throughout the two years; the percentage of standing trees harboring bugs was 5.60% with a 95% confidence interval of (3.27%, 7.93%) (n = 375). No overwintering adults were found from downed trees; a 95% confidence interval for this zero estimation is (0%, 0.61%) (n = 605). Thus, the estimated proportion of standing trees harboring *H. halys* was significantly greater than that of downed trees (Fisher's exact test; P<0.001). Second, those standing trees harboring *H. halys* had DBH >19.0 cm with a mean and standard error of 36.5±4.1 cm. Lastly, *H. halys* were primarily recovered from oak (*Quercus* spp.) and locust (*Robinia* spp.) (Fig. 3). These two species were dominant in the study sites, consisting of 65.5% of identifiable dead trees. *H. halys* were found beneath loose thick bark and between tight crevices present in decomposing woody tissue. The percentage of dead trees that were standing, with DBH >19.0 cm, and oak or locust was 11.8% among all strip transect sample sites. That is, these trees have favorable characteristics that *H. halys* are likely to select and utilize as overwintering sites in forested landscapes.

A total of 104 dead trees which shared the aforementioned three characteristics were selectively chosen and sampled over the two seasons. Among those selected trees, 36.0% and 27.8% of the trees harbored overwintering *H. halys* in 2011–2012 and 2012–2013, respectively, averaging 31.7% across the two years. There was no significant difference in the proportions of trees harboring *H. halys* between the two years (*χ* ^2^ = 0.81, d.f. = 1, P = 0.368). The success rate of locating *H. halys* among the selected trees (31.7%) was significantly higher than the success rate of the random samples (1.6%) (*χ* ^2^ = 104.39, d.f. = 1, P<0.001), verifying that the established characteristics can be used to identify likely overwintering sites of *H. halys*. The mean numbers (± SE) of *H. halys* from trees harboring bugs were 6.61±2.15 and 5.00±1.98 in 2011–2012 and 2012–2013, respectively, when ∼20% of the surface area of standing trees was sampled. There was no significant difference in the mean *H. halys* numbers between the two years (*χ* ^2^ = 0.10, d.f. = 1, P = 0.751).

### Leaf litter samples by human surveyors

No *H. halys* were found from 240 leaf litter samples collected over the two seasons. Six native pentatomid species were found overwintering in leaf litter. *Chinavia hilaris* (Say) was the dominant species recovered from 20 samples, followed by *Euschistus servus* (Say) (2 samples), *Podisus maculiventris* (Say) (2 samples), *Menecles insertus* (Say) (2 samples), *Euschistus tristigmus* (Say) (1sample), and *Murgantia histrionica* (Hahn) (1 sample). In all cases where stink bugs were found in the samples, the density of bugs was one per sample across species found.

### Laboratory and semi-field detector canine studies

**Figure 4:**
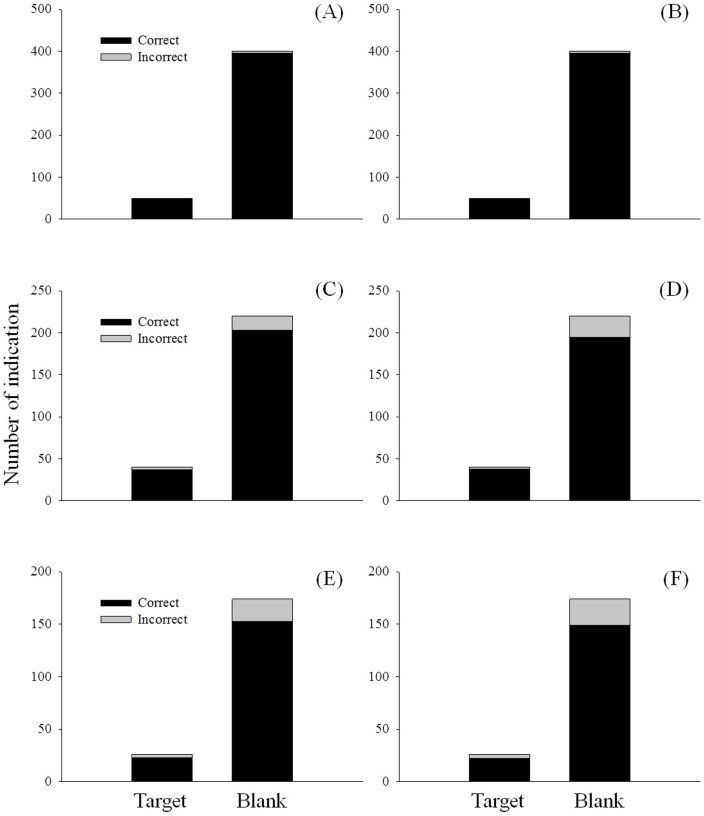
Number of correct and incorrect indications by detector canines in the trials to locate *Halyomorpha halys* concealed in cardboard boxes for Canine 1 (A) and Canine 2 (B), concealed on the ground for Canine 1 (C) and Canine 2 (D), and concealed on the live trees for Canine 1 (E) and Canine 2 (F).

In the laboratory experiment in which odor point sources were concealed in boxes, both canines accomplished 100% proficiency for *H. halys* target odor and 98.8% proficiency for blank control (Figs. 4A & B). In the first semi-field experiment in which odor point sources were concealed on the ground, Canine 1 and Canine 2 demonstrated 92.5% and 95.0% proficiency at locating *H. halys* target and 92.3 and 88.6% proficiency on the blank control, respectively (Figs. 4C & D). It took on average 3.5 min for a detector canine to examine 20 odor point sources on the ground. In the second semi-field trial in which odor point sources were concealed on live peach trees, Canine 1 and Canine 2 demonstrated 88.5% and 84.6% proficiency on *H. halys* target and 87.9 and 85.6% proficiency on the blank, respectively (Figs. 4E & F). It took on average 5.8 min for a detector canine to examine 20 odor point sources on the peach trees.

### Detection of *H. halys* by canines in natural landscape

Responses by detector canines to dead trees were analyzed in relation to the tree characteristics of potential *H. halys* overwintering sites identified previously by human surveyors (Table 2). The results of this study indicate that the responses made by detector canines and the tree characteristics indentified by human surveyors can complement each other, leading to more proficient detection of overwintering *H. halys*. Among 187 dead trees found in the study site, detector canines exhibited positive indications to 11 trees. Two of them had the favorable characteristics as overwintering sites indentified by human surveyors. Indeed, *H. halys* were recovered only from those two trees in which both the canines indicated the presence of the insects and shared the three key characteristics. The other nine trees, indicated by the canines, did not have the favorable characteristics; subsequent sampling confirmed that the insects were not present. Detector canines showed no indication to 176 out of 187 dead trees over the study. When 50 of those trees were randomly sampled, no *H. halys* were found from them and most of those trees (96.0%) did not meet the established tree characteristics. It took on average 6.2 min for a canine to survey a 500-m^2^ transect with ∼15 dead trees.

**Table 2:** Detection of *Halyomorpha halys* by detector canines from dead trees in the natural landscape.

Positive indication by detector canines^1^	Favorable tree characteristics^2^	No. of trees sampled^3^	No. of trees verified with *H. halys*
Yes	Presence	2	2
Yes	Absence	9	0
No	Presence	2	0
No	Absence	48	0

1 Yes refers that canines indicated the presence of *H. halys*; No refers that canines indicated the absence of *H. halys*.

2 Dead trees with the favorable characteristics (standing, DBH >19 cm, oak or locust) were identified as likely overwintering sites of *H. halys* based on the results from human surveyors.

3 All trees indicated by canines were destructively sampled during the survey (n = 11). For trees that canines did not show positive indications (n = 176), 50 of them were randomly selected and destructively sampled after the survey.

## Discussion

Very few attempts have been made to characterize overwintering ecology of *H. halys* in natural landscapes [2]. Knowledge of overwintering ecology of *H. halys* in natural landscapes has been based only on anecdotal and isolated observations from its native range [11], [18], [19]. Neither preferred overwintering habitat types nor the abundance of *H. halys* overwintering in those habitats has been established through rigorous sampling in natural landscapes. The results of this study reveal that there are evident preferences by *H. halys* for selection of overwintering structures in natural landscapes. In deciduous forests, *H. halys* overwinter in dead, standing trees with thick tree bark which provide protective crevices for shelter, mostly oak (*Quercus* spp.) and locust (*Robinia* spp.). In contrast, no overwintering *H. halys* were found from downed trees or leaf litter, although other native pentatomid species were reported overwintering in ground litter in South Carolina [21] and also recovered in this study.

It is noteworthy that overwintering *H. halys* were found almost exclusively from dry surfaces within dead, standing trees. Indeed, tree tissues from downed dead trees had 2.4-fold higher moisture content compared with standing dead trees (Lee, D.-H., unpublished data). Therefore, the results strongly suggest that *H. halys* preferentially use dead, standing trees to overwinter and seem to avoid wet conditions present in downed trees and leaf litter on the ground. This preference for cool, dry locations is also found in human-made structures [9]. In a laboratory setting, antennectomy of adult *H. halys* prevented the insects from forming overwintering aggregations [23]. This result suggests that *H. halys* use chemical or tactile cues within a short range to form the aggregations and potentially use the cues to select dry surfaces.

Our results indicate that dead, standing oak or locust trees with >19-cm DBH are likely to harbor overwintering *H. halys* in the forested areas in the mid-Atlantic region surveyed. Our data indicate that 12% of total dead trees share these favorable traits as potential overwintering sites. We also found that the mean density of *H. halys* was 5.9 adults among the trees harboring *H. halys* from samples comprising ∼20% of the total tree surface area. This mean density should be considered a conservative estimate as sampling was done only from accessible outer parts of tree trunks due to logistical limitations. At present, limited information is available on the abundance of overwintering *H. halys* inside inner parts of tree trunks (e.g., crevices between decomposing woody tissues). Continuous efforts are needed to conduct more thorough destructive sampling targeting the inner parts of trees and apply other methods such as inducing overwintering *H. halys* to emerge from those concealed structures in a warm setting.

There was no significant difference in the proportion of dead trees harboring *H. halys* or the mean number of the adults per tree between the two seasons. Interestingly, *H. halys* adult density captured in traps during the late season (e.g., late September through early October) around the study sites was ∼60% higher in 2012 than 2011 [24]. Anecdotal evidence indicates that the level of overwintering populations dispersing into human-made structures were substantially higher in 2012 than 2011 as well. However, our data show that this was not the case for *H. halys* populations overwintering in natural landscapes. Further studies are warranted to examine what factors govern the selection of *H. halys* overwintering sites between natural and human-made overwintering sites and how this may influence the overall population growth of *H. halys* at longer terms.

*H. halys* populations overwintering in dead, standing trees are patchily distributed across the forested landscapes and highly concealed in the structures. Populations were found in both small woodlots directly adjacent to agricultural production and forested areas >2 km from agricultural production. This distribution pattern is different from highly concentrated and often noticeable populations overwintering in human-made structures. Therefore, detection and management is likely more difficult for overwintering populations in natural landscapes compared with those in human-made structures leading to a higher probability of *H. halys* populations overwintering in natural landscapes to remain unchecked. Given that adult *H. halys* has a wide host range [2] and the capacity to disperse over >2-km distance [20], *H. halys* populations may first utilize diverse wild hosts (e.g., mulberry, elm and willow) after spring emergence in forested areas and subsequently invade into agricultural production [11]. Wooded areas cover large portions of landscapes in the invaded regions in which *H. halys* have established as a serious agricultural pest (e.g., 78% forested land in West Virginia). Therefore, it is critical to evaluate the threat posed by *H. halys* overwintering in natural landscapes and potentially develop preemptive management programs in order to disrupt the establishment of *H. halys* populations from overwintering sites to cultivated crops.

In this study, we report the promising potential of using detector canines to locate highly dispersed and concealed *H. halys* populations overwintering in natural landscapes. Domestic canines discriminate and detect specific odors exceptionally well [25], and this unique ability has been utilized over the years to detect various materials including drugs, explosives, and agricultural quarantine items. Wallner and Ellis [26] first reported the use of canines to locate egg masses of the gypsy moth, *Porthetria dispar* (L.). Since then, canines have been used to detect insect pests including screwworms, *Cochliomyia hominivorax* (Coquerel) [27], termites, *Reticulitermes flavipes* (Kollar) [28], and bed bugs, *Cimex lectularius* (L.) [29]. Our data indicate that responses of detector canines in combination with the key tree characteristics of *H. halys* overwintering sites identified by human surveyors can complement each other leading to more accurate and fast detection of overwintering *H. halys* in natural landscapes. In particular, it is noteworthy that overwintering *H. halys* were recovered only from dead, standing trees indicated by both the canines and those favorable tree characteristics established by human surveyors.

Among 187 dead trees surveyed, the canines showed nine false positive indications to trees that did not meet the established characteristics of overwintering sites. Observational data indicates that these incorrect indications by detector canines could be due to responses to *H. halys* in other nearby trees located upwind from the indicated trees; many of these peripheral trees had favorable overwintering characteristics. Indeed, sustained winds during the survey likely led to mixing of odors throughout the survey area, leading to those indications by the canines. In most cases, however, this type of response was immediately recognized by canine trainers as different from correct indications typically leading to pinpointing of the targets. Thus, combined use of the detector canines and indentified tree characteristics will allow us to execute a fast and reliable scanning process to select only highly likely overwintering structures of *H. halys* in the field.

Due to economic and logistical constraints in procuring and training detector canines, this study included only two canines for the *H. halys* detection program. Because the variability among canine individuals in detecting *H. halys* was beyond the scope of this study, we used two detector canines which had already passed a procuring standards established at the NDDTC. These canines were subsequently trained for a new target odor, *H. halys*. Thus, it should be considered that this study cannot address variance among canines. With this caveat, the two canines demonstrated consistent performance of locating *H. halys* targets in various laboratory trials and in natural environments.

Overwintering is a pivotal period within the lifecycle of insects that can directly affect the abundance and distribution of pest populations in agroecosystems over the growing season [30]. However, the study of this important stage has been neglected in many cases [30]. Understanding the overwintering ecology of *H. halys* is critical and the information should be incorporated into the integrated pest management programs against this destructive invasive species, such as deployment strategies for monitoring traps and biological control agents. The results of this study revealed the characteristics of overwintering structures used by *H. halys* in natural landscapes and the relative pest abundance in those structures. This information is fundamental for establishing risk levels for *H. halys* populations at geographical scales, in conjunction with the knowledge of human-made overwintering sites. Detector canines can substantially improve the accuracy and efficiency of sampling efforts on overwintering *H. halys* populations in natural landscapes. Further studies are warranted to elucidate the survivorship and spring emergence patterns of overwintering *H. halys* from natural overwintering sites, as well as human-made structures. These efforts will help us quantify the contribution of *H. halys* populations from various overwintering sites to pest pressure and accordingly develop site-specific management/monitoring strategies by targeting overwintering stage of *H. halys*.

## Supporting Information

Video S1 **Laboratory detection of** ***Halyomorpha halys*** **by canines.**
(MPEG)
